# Modern Indulgences

**Published:** 1889-07

**Authors:** E. Johnson


### MODERN INDULGENCES.

The indulgences wherewith even young and healthy men indulge themselves—"the comforts" as they call them, are infinifely worse than absurd; because the opposites of all these, so far from being injurious to health, are absolutely necessary to it. We actually kill ourselves with "comforts." It is absolutely digusting to see the excessive care and caution with which great fellows with rough beards on their chins, and with fists large and strong enough to fell an ox, and legs long enough to bestride the Thames—I say it is neither more than disguisting to see these lackadaisical women in the likeness of men, or rather these monsters, which are neither men nor women—I say it is *literally disgusting*, and degrading, too—degrading to our nature, to our being—degrading to the physical energies of nature's masterwork—to see the care and painstaking with which these abortive monstrosities, the progeny of a morbid and excessive refinement, protect their delicate and precious persons from a few drops of rain or a little mist, or a little unusual inclemency of the weather, of whatever kind.

I got into a coach the other day, because there was no room outside, the weather was dry but cold and sharp. In the corner of the coach there sat a mighty combination of bone, muscle, thew and sinew, all assisting in the formation of what should have been a man. He was at least six feet high and "bearded like the pard;" and seemed as well able to carry the coach as the coach was to carry him. As soon as I entered the vehicle, I let down the window; but before I had quite succeeded in doing so, there issued from amidst the cloaks, and coats, and shawls, and wrappings and mufflings, in which this great thing had enveloped itself, a voice of supplication and woe; "For God's sake don't let the window down! I am *so susceptible—extremely susceptible*!" Look at the delicate and fragile plant in your garden! see how it is buffeted by the wind, and alternately scorched by the sun, and deluged by the rain, and frozen by the frost, and spattered by the mud, and bruised by the passenger's foot! Yet how greenly and heartily it grows. Take it into your parlor and warm it by the fire, and curtain it with flannel, and defend it from the cold, and the wind and the rain, and rude contact of the traveller's foot, and the other "discomforts" of its outdoor existence. What think you? Will it continue to flourish as greenly as before?—Dr. E. Johnson.

